# Factors associated with cesarean birth in nulliparous women: A multicenter prospective cohort study

**DOI:** 10.1111/birt.12654

**Published:** 2022-06-13

**Authors:** Sunita Panda, Cecily Begley, Paul Corcoran, Deirdre Daly

**Affiliations:** ^1^ School of Nursing and Midwifery Trinity College Dublin Dublin Ireland; ^2^ National Perinatal Epidemiology Centre University College Cork Cork Ireland

**Keywords:** antenatal care, breech presentation, cesarean birth, epidural, induction of labor, infertility, labor management

## Abstract

**Background:**

There is widespread concern around the rising rates of cesarean births (CBs), especially among first‐time mothers, despite evidence suggesting increased morbidities after birth by cesarean. There are uncertainties around factors associated with rising rates of CBs among first‐time mothers in Ireland, and insight into these is essential for understanding the rising trend in CBs. Therefore, this study aimed to identify the factors associated with CBs in nulliparous women.

**Methods:**

A prospective cohort study was conducted in three maternity hospitals in the Republic of Ireland between 2012 and 2017. Data were collected from 3047 nulliparous women using self‐administered surveys antenatally and at 3 months postpartum and from consenting women’s hospital records (n = 2755) and analyzed using the Poisson regression to assess associations between demographic and clinical factors and the main outcome measures, planned and unplanned CBs.

**Results:**

Common risk factors for planned and unplanned CBs were being aged ≥40 years, being in private care, multiple pregnancy, and fetus in breech or other malpresentations. An unplanned CB occurred for 22.43% (n = 377/1681) of women who did not have induction of labor (IOL) or who had IOL with no epidural, but the risk was about twice as high for women who had IOL and epidural.

**Conclusions:**

Findings confirm multifactorial reasons for CB and the challenge of reversing the increasing CB rate if maternal age, overweight/obesity, infertility treatment, multiple pregnancy, and preexisting hypertension in Ireland continue to increase. There is a need to address prelabor interventions, especially IOL combined with epidural analgesia with respect to unplanned CB.

## 1 INTRODUCTION

Internationally, rates of cesarean birth (CB) continue to rise despite universal consensus that such frequent use of CB cannot be medically justified.^1^ In Europe, rates vary from as low as 16.1% (Iceland) to as high as 56.9% (Cyprus), with 27 countries at a CB rate above 20%.^2^ Analysis of data from 121 countries indicated a relative annual increase of CB by 4.4% between 1990 (6.7%) and 2014 (19.1%).^1^ CB rates in the Republic of Ireland, although in the European average range, rose from 27% in 2010^2^ to the latest rates at 34.3% in 2019,^3^ a 27% increase. There are unexplained variations in rates between the 19 maternity units in Ireland^4^ with a wide variation from 27% to 41%,^3^ and a rate of 36.1%^3^ for first‐time mothers; more than half of first‐time mothers giving birth by cesarean in one of the maternity units.^3^ Ireland is a small country, with a homogeneous population, and the highest CB rates are not seen in the larger urban sites where women with more complex needs may attend. Previous Irish research has shown clearly that first‐time mothers attending a consultant obstetrician privately are more than twice as likely to have a CB, despite the fact that these women do not have a higher at‐risk profile.^5^ In Turner et al’s study, the higher rates of CB were attributed to women choosing elective CB, being reluctant to take risk, and being able to afford health insurance to choose continuity of care from a senior obstetrician.^6^

Rising rates of CB among first‐time mothers (nulliparous women) raise concerns because of the added risk of repeat CBs in subsequent pregnancies, and with evidence suggesting an increased risk of short‐term or long‐term complications after CB for mothers and babies compared with vaginal births.^7^, ^8^

Several factors are associated with the rising rates of CB. An increase in CB among nulliparous women has been associated with increasing risk factors such as advanced maternal age, obesity^9^, previous treatment for infertility and hypertension/pregnancy‐induced hypertension,^10^ or maternal age.^11^ There is consensus around clinical reasons for CB, such as labor dystocia, fetal distress, and acute clinical emergency (eg, severe antepartum hemorrhage or umbilical cord prolapse).^12^ Although fetal breech presentation continues to be a leading clinical reason to perform CBs, there is a strong emphasis on the use of external cephalic version (ECV) to reduce the rate.^13^ Despite evidence around the positive impact on reducing rates of CB by reducing the number of inductions of labor (IOL),^14^ this continues to be another major factor contributing to high rates of CB.^15^ There is mixed evidence around most of these factors such as maternal characteristics (age and BMI),^9^ labor interventions (eg, IOL),^15^ and systems‐level characteristics (eg, private health care);^5^, ^6^ thus, it was essential to confirm the findings from Irish data. It is important to replicate these results in the Irish context to, first, improve maternity care within Ireland through advancing knowledge by exploring clinical scenarios, and to second, add additional evidence and revisit policies, which in turn could have implications for whether efforts to reduce CB might be generalizable from one country to another. It is also essential to acknowledge that factors that lead to CBs in first‐time mothers influence decision‐making for the future and subsequent mode of births.^16^ Therefore, the objective of this study was to identify the combination of prepregnancy, pregnancy, and intrapartum factors, nonclinical and clinical, and possible patterns, associated with CB in nulliparous women in the Republic of Ireland.

## 2 METHODS

**FIGURE 1:**
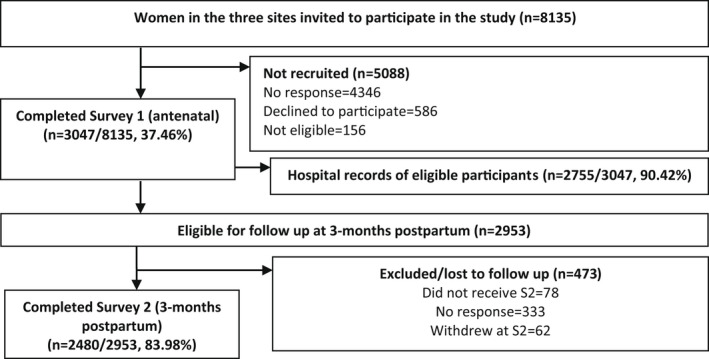
Participant flowchart of recruitment and response rates

**FIGURE 2:**
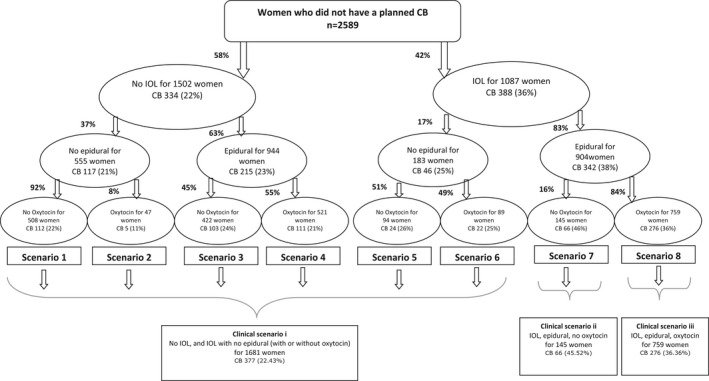
Clinical scenarios and the risk of unplanned cesarean birth

This prospective cohort study was conducted after ethics approval from the university and the three study sites in the Republic of Ireland between 2012 and 2017. The population in these settings included women from urban and rural areas, with both high and low obstetric and medical risks. All eligible nulliparous women aged 18 years or older, who could read and understand English, received the study information from the midwives at the first antenatal booking clinics and verbally consented to be contacted by the researcher 1‐2 weeks later to answer their questions. Women who were willing to participate were asked to complete and post the antenatal survey and the consent form using the addressed envelope. Women were followed up by a phone call and text message. The participant flowchart presents the retention and response rates up to 3 months postpartum (Figure 1). Data were collected using the Maternal health And Maternal Morbidity in Ireland (MAMMI) study questionnaires (available at https://www.tcd.ie/mammi/gdpr.html), which gathered information on women’s prepregnancy and early pregnancy health antenatally and at 3, 6, 9, and 12 months postpartum. The MAMMI study questionnaires had been tested for face and content validity, and reliability, and then piloted.^17^ Data storage and processing complied with the European Data Protection regulation, General Data Protection Regulations (GDPR) 2018 (https://gdpr‐info.eu/ ). A total of 3047 women were recruited to the study. Data from women who had late miscarriages or fetal deaths were excluded from the analysis. The questionnaire data were merged with the hospital records data (completed by clinicians) from consenting women after checking and cleaning of the individual databases. Preparation of the data involved spot checking (10% of the total surveys), thorough cleaning of variables, recoding (into binary variables), recategorizing (eg, into three BMI categories), and creating new variables (eg, creating clinical scenarios outlined in Figure 2).

Women attending for all types of maternity care at the site hospitals were included in the study. There are three maternity care packages available to women at two study sites (public, semiprivate, and private maternity care), and two packages at one of the study sites (public and private maternity care). Public care is free to all women who are residents in the Republic of Ireland. Women who choose semiprivate and private care pay a fee, which is not covered by their private health insurance. In the public and semiprivate system, women book for their maternity care in their chosen maternity unit, and their care during pregnancy, intrapartum, and postpartum period is provided by midwives and the team of obstetricians. During pregnancy, the care is shared between the hospital and general practitioners (GPs). Women who choose private care can book directly with a consultant obstetrician in their chosen maternity unit, and the consultant obstetrician is directly responsible for decision‐making and their care.

### 2.1 Statistical analyses

Of the total number of women recruited to the study (n = 3047), women who had completed the antenatal survey and consented for access to their hospital records (n = 2755/3047, 90.4%) were included in the analysis. Prepregnancy factors (maternal age, prepregnancy BMI, medical conditions such as any diabetes, hypertension, and asthma, and treatment for infertility) sourced from the antenatal survey, pregnancy factors (type of care, number of fetus[es], gestational age at birth, and presentation of fetus at birth), and intrapartum factors (IOL, IV oxytocin in labor, and epidural for pain management) sourced from hospital records of consenting women were identified from literature and included in the analysis to explore possible associations with planned and unplanned CB. Data for the variables were obtained either from the questionnaire or from the medical record, but not both. Mode of birth was the outcome (dependent) variable in this analysis.

Sample representativeness was assessed by comparing data on socio‐demographic characteristics and pregnancy and birth details with routinely collected national perinatal data for all women^2^ and data for nulliparous women^3^ giving birth in the 19 maternity units in the Republic of Ireland during the study period.

Descriptive and inferential statistics were used to assess the factors associated with CB using the IBM statistical software SPSS version 24. Socio‐demographic characteristics (of all women in the study (n = 3047)) and pregnancy and birth details (from hospital data (n = 2755)) were analyzed using descriptive statistics. Prepregnancy, pregnancy, and intrapartum factors associated with CB were analyzed first using chi‐square tests. Factors with statistically significant associations, that is, a *P*‐value <0.05, were included in multivariable Poisson regression models. Associations are presented as adjusted risk ratios (ARRs) with 95% confidence intervals (95% CIs). In assessing the risk of CB, we considered the interaction between three common intrapartum interventions, “IOL,” “epidural for pain management in labor,” and “use of IV oxytocin in labor.” Associations are presented to reflect the observed evidence of interaction between these interventions and risk of CB.

Because women with multiple gestation, preterm gestation, and malpresentation have different care pathways to CB, we conducted multivariable Poisson regression models both including and excluding these women, in order to demonstrate that the findings were robust.

## 3 RESULTS

### 3.1 Socio‐demographic background and pregnancy and birth details of women in the study

The socio‐demographic background of all women recruited to the study (n = 3047), and pregnancy and birth details of women whose data were included in the analysis (n = 2755) are presented in Table 1 (Table S1—Additional socio‐demographic characteristics). Study participants were representative of socio‐demographic characteristics of women birthing in Ireland,^18^ that is, being Irish by birth and married, singleton pregnancy, IOL and spontaneous vaginal birth (SVB), and CB. However, women aged up to 24 years and ≥ 40 years are slightly underrepresented, and women aged 35‐39 years overrepresented. In relation to rates of epidural for pain management in labor, women in the study (nulliparous) from all three study sites were, understandably, not representative when compared to the national statistics, which presents data on all women and not for nulliparous and multiparous women separately (Table 1).

**TABLE 1:** Study participant characteristics compared with national data

	Socio‐demographic characteristics	Study participants (all nulliparous)		Perinatal statistics report^18^	
		Frequency	(%)	Frequency	(%)
Maternal age	Up to 24 years	239	7.8	6327	9.9
	25‐29 years	620	20.4	11 431	17.8
	30‐34 years	1316	43.2	23 078	36
	35‐39 years	734	24.1	18 829	29.4
	≥40 years	138	4.5	4133	6.4
	Total	3047		64 097 (all parities)	
Region of birth	Irish	2106	70.2	48 937	76.3
	Europe (excluding Ireland and the United Kingdom)	568	18.9	8761	13.7
	United Kingdom	137	4.6	1463	2.3
	America	44	1.5	773	1.2
	Asia	77	2.6	2344	3.7
	Africa	53	1.8	1445	2.7
	Australia and other Oceania	14	0.5	81 (Australia) and 269 (not stated)	0.5
	Total	2999	100	64 097 (all parities)	100
Relationship status	Married	1828	61.0	39 882	62.2
	Single	92	3.1	23 301	36.4
	In relationship with or without partner	1046	34.9	21	‐
	Other (divorced, widowed, separated)	30	1.0	877	1.4
	Total	2996	100	64 097 (all parities)	100
		Study participants		Irish Maternity Indicator System^3^	
Number of fetus(es)	Singleton gestation	2700	98.0	57 204	98.2
	Multiple gestation	55	2.0	1068	1.8
	Total	2755	100	58 272 (all parities)	100
Induction of labor	No	1655	60.3	13 330	59
	Yes	1089	39.7	9261	41
	Total	2744	100	22 591(nulliparous)	100
Epidural for pain management in labor	No	566	26.7	34 627	59.4
	Yes	1554	73.3	23 645	40.6
	Total	2120	100	58 272 (all parities)	100
Mode of birth	Spontaneous vaginal birth	926	33.6	8343	36.9
	Assisted vaginal birth	941	34.7	6083	26.9
	Planned cesarean	166	6.0	8165	36.1
	Unplanned cesarean birth	722	26.2		
	Total	2755	100	22 591(nulliparous)	100

Data were missing for country of birth (n = 48), relationship status (n = 51), induction of labor (n = 11), and epidural for pain management in labor (n = 635). Table S1 and S2 present additional socio‐demographic characteristics and pregnancy and birth details of participants. The Perinatal Statistics Report does not report all variables by parity and these are presented for nulliparous and multiparous women when available.

### 3.2 Mode of birth

A total of 90.4% of the cohort of women recruited to the study had consented for access to their medical records (n = 2755/3047), and of these, approximately one‐third (888/2755, 32.2%) had a CB (planned n = 166, 6% and unplanned n = 722, 26.2%) similar to the CB rates for nulliparous women from the three study sites.^19^, ^20^, ^21^ Compared with the national statistics, women in the study were representative of SVBs and CBs (planned and unplanned) and overrepresentative of assisted vaginal births (AVBs) (Table 1) (Table S2—Additional pregnancy and birth details). The most common reason for a planned CB was fetal breech presentation (n = 74/166, 44.6%) and for an unplanned CB was fetal distress (n = 337/722, 46.7%). Macrosomia (13/166, 7.8%) and fetal growth restriction (7/166, 4.2%) accounted for a further 12% of planned CBs (Table S3—Reasons for planned and unplanned CB).

### 3.3 Factors associated with risk of planned CB

Separate consideration of each prepregnancy and pregnancy factor showed that maternal age, treatment for infertility, type of care, multiple gestation, malpresentation, and preterm gestation were associated with risk of planned CB (Table 2). When these factors were considered together, risk of planned CB was increased for women aged ≥40 years (ARR 2.77, 95% CI 1.52‐5.05, *P* = 0.001), having had treatment for infertility (2.03, [1.38‐2.99], *P* < 0.001), being in semiprivate (1.92, [1.33‐2.78], *P* = 0.001) or private care (2.78, [1.92‐4.04], *P* < 0.001), and having a multiple gestation (2.81, [1.48‐5.35], *P* < 0.05) and a breech or other malpresentations (15.99, [11.77‐21.74], *P* < 0.001) (Table 2). When women with multiple gestation, preterm gestation, or malpresentation were excluded, the associations were similar, but the effects of age were stronger; for example, ARR for women ≥40 years of age was 6.13 (2.06‐18.27, *P* = 0.001).

**TABLE 2:** Factors associated with the risk of planned CB

Factor	Group	Planned CB (n = 166)	Other modes (n = 2589)	ARR (95% CI)	*P*‐value
Maternal age	Up to 24 years	6 (2.8%)	207 (97.2%)	0.63 (0.26‐1.53)	0.306
	25‐29 years	26 (4.7%)	524 (95.3%)	1 (Ref)	
	30‐34 years	58 (4.9%)	1132 (95.1%)	1.00 (0.63‐1.59)	0.984
	35‐39 years	52 (7.7%)	623 (92.3%)	1.42 (0.88‐2.30)	0.151
	≥40 years	24 (18.9%)	103 (81.1%)	2.77 (1.52‐5.05)	0.001
Prepregnancy BMI	Ideal weight	107 (6.0%)	1678 (94.0%)	1	
	Overweight	31 (6.3%)	458 (93.7%)	1.00 (0.67‐1.50)	0.987
	Obese/very obese	16 (6.0%)	252 (94.0%)	1.04 (0.62‐1.77)	0.876
	Missing	12 (5.6%)	201 (94.4%)	1.01 (0.54‐1.89)	0.983
Treatment for infertility	No	124 (5.1%)	2328 (94.9%)	1 (Ref)	
	Yes	41 (14.0%)	253 (86.0%)	2.03 (1.38‐2.99)	<0.001
Type of care	Public	77 (4.3%)	1718 (95.7%)	1 (Ref)	
	Semiprivate	45 (7.8%)	529 (92.2%)	1.92 (1.33‐2.78)	0.001
	Private	44 (11.4%)	342 (88.6%)	2.78 (1.92‐4.04)	<0.001
Number of fetus(es)	Singleton gestation	154 (5.7%)	2546 (94.3%)	1 (Ref)	
	Multiple gestation	12 (21.8%)	43 (78.2%)	2.81 (1.48‐5.35)	0.002
Cephalic presentation	Cephalic	91 (3.5%)	2528 (96.5%)	1 (Ref)	
	Breech or other malpresentations	75 (55.2%)	61 (44.9%)	15.99 (11.77‐21.74)	<0.001
Gestational age at birth	Term	148 (5.7%)	2446 (94.3%)	1 (Ref)	
	Preterm	18 (11.2%)	143 (88.8%)	1.17 (0.69‐2.00)	0.554

*Note*: Data were missing for treatment for infertility (n = 9). The model included prepregnancy factors (maternal age, prepregnancy BMI, and treatment for infertility. Preexisting medical conditions were not significantly associated with the risk of a planned CB in bivariable analysis, and hence were not included in the multivariable Poisson regression model. Prepregnancy BMI was not identified to be significantly associated with the risk of planned CB, but it was included in the model because of its clinical importance) and pregnancy factors (type of care, number of fetus(es), gestational age, and presentation of fetus at birth).

Abbreviations: ARR, adjusted risk ratio; CI, confidence interval.

### 3.4 Factors associated with risk of unplanned CB

Bivariate analysis of each prepregnancy, pregnancy, and intrapartum factor showed that maternal age, prepregnancy BMI, treatment for infertility, hypertension, diabetes, asthma, type of care, multiple gestation, malpresentation, preterm gestation, IOL, and epidural were associated with risk of unplanned CB (Table 3). When considered together, the risk of unplanned CB was increased for women aged ≥40 years (1.76, [1.25‐2.46], *P* < 0.001), and those who were overweight (1.58 [1.32‐1.90], *P* < 0.001) or obese/very obese (1.29 [1.02‐1.64], *P* < 0.05), with preexisting hypertension (1.41 [95% CI] 1.03‐1.92, *P* < 0.05) or asthma (1.22 [1.02‐1.47], *P* < 0.05), in private care (1.33 [1.07‐1.64], *P* < 0.010), and with multiple gestation (1.56 [1.01‐2.40], *P* = 0.045), preterm gestation (1.68 [1.27‐2.23], *P* < 0.001), and breech or other malpresentations of the fetus at birth (4.54 [3.38‐6.09], *P* < 0.001). There was an interaction between IOL and epidural for pain management in labor whereby the risk of unplanned CB was increased for those with IOL (without epidural) (1.57 [1.10‐2.23], *P* < 0.05) and for women with epidural (without IOL) (1.28 [1.02‐1.62], *P* < 0.05), but the risk was more than doubled for women with both IOL and epidural (2.14 [1.71‐2.69], *P* < 0.001). Women aged up to 24 years had less risk of an unplanned CB (0.59 [0.39‐0.90], *P* = 0.014). The above associations were very similar in the analysis that excluded women with multiple gestation, preterm gestation, or malpresentation.

**TABLE 3:** Factors associated with the risk of unplanned CB

Factors		Unplanned CB (n = 722)	Vaginal births (n = 1867)	ARR (95% CI)	*P*‐value
Maternal age	Up to 24 years	29 (14.0%)	178 (86.0%)	0.59 (0.39‐0.90)	0.014
	25‐29 years	127 (24.2%)	397 (75.8%)	1 (Ref)	
	30‐34 years	292 (25.8%)	840 (74.2%)	1.00 (0.81‐1.24)	0.992
	35‐39 years	217 (34.8%)	406 (65.2%)	1.21 (0.96‐1.53)	0.115
	40 years and over	57 (55.3%)	46 (44.7%)	1.76 (1.25‐2.46)	<0.001
Prepregnancy BMI	Ideal weight	403 (24.0%)	1275 (76.0%)	1 (Ref)	
	Overweight	178 (38.9%)	280 (61.1%)	1.58 (1.32‐1.90)	<0.001
	Obese/very obese	90 (35.7%)	162 (64.3%)	1.29 (1.02‐1.64)	0.034
	Missing	51 (25.4%)	150 (74.6%)	1.12 (0.82‐1.52)	0.481
Hypertension *M* = 41	No	664 (27.1%)	1783 (72.9%)	1 (Ref)	
	Yes	46 (45.5%)	55 (54.5%)	1.41 (1.03‐1.92)	0.030
Any diabetes *M* = 43	No	704 (27.8%)	1831 (72.2%)	1 (Ref)	
	Yes	7 (63.6%)	4 (36.4%)	1.70 (0.80‐3.6)	0.169
Asthma *M* = 28	No	568 (26.6%)	1567 (73.4%)	1 (Ref)	
	Yes	147 (34.5%)	279 (65.5%)	1.22 (1.02‐1.47)	0.034
Treatment for infertility *M* = 8	No	618 (26.5%)	1710 (73.5%)	1 (Ref)	
	Yes	102 (40.3%)	151 (59.7%)	1.00 (0.79‐1.27)	0.973
Type of care	Public	442 (25.7%)	1276 (74.3%)	1 (Ref)	
	Semiprivate	150 (28.4%)	379 (71.6%)	1.05 (0.87‐1.28)	0.603
	Private	130 (38.0%)	212 (62.0%)	1.33 (1.07‐1.64)	0.010
Number of fetus(es)	Singleton gestation	695 (27.3%)	1851 (72.7%)	1 (Ref)	
	Multiple gestation	27 (62.8%)	16 (37.2%)	1.56 (1.01‐2.40)	0.045
Gestational age at birth	Term	650 (26.6%)	1796 (73.4%)	1 (Ref)	
	Preterm	72 (50.3%)	71 (49.7%)	1.68 (1.27‐2.23)	<0.001
Presentation of fetus at birth	Cephalic	664 (26.3%)	1864 (73.7%)	1 (Ref)	
	Breech and other malpresentations	58 (95.1%)	3 (4.9%)	4.54 (3.38‐6.09)	<0.001
Use of IOL and epidural *M* = 3	Neither	117 (21.1%)	438 (78.9%)	1 (Ref)	
	IOL only	46 (25.1%)	137 (74.9%)	1.57 (1.10‐2.23)	0.013
	Epidural only	215 (22.8%)	729 (77.2%)	1.28 (1.02‐1.62)	0.035
	Both	342 (37.8%)	562 (62.2%)	2.14 (1.71‐2.69)	<0.001

*Note*: Use of IV oxytocin was not statistically significantly associated with unplanned CB in bivariable analysis and, thus, was not included in multivariable analysis. Prepregnancy BMI was not significantly associated with CB on bivariable analysis; however, it was included in multivariable regression models because of its clinical importance.

Abbreviation: M, missing.

### 3.5 Clinical scenarios associated with the risk of unplanned CB

The frequency of IOL, epidural, and oxytocin is detailed in Figure 2. Of the 2589 women who did not have a planned CB, 42.0% (n = 1087) had their labor induced. The most common reasons for IOL were post‐term gestation (341/1087, 31.4%), prolonged rupture of membranes (242/1087, 22.3%), and pregnancy‐induced hypertension/preeclampsia (109/1087, 10.0%) (Table S4). Over one‐third of the women whose labor was induced had an unplanned CB (388/1087, 35.7%), whereas this was the case for 22.2% (334/1502) of the women with no IOL.

No significant difference was observed in the risk of unplanned CB between Scenarios 1 and 6, which represent women who did not have IOL or who had IOL without epidural. Considered together, 1681 women (1681/2589, 64.9%%) were in this low intervention clinical scenario (clinical scenario i) and 22.4% (377/1681) of them had an unplanned CB. In contrast, risk of an unplanned CB for women who had an IOL and epidural was 45.5% (66/145) if they did not have IV oxytocin (clinical scenario ii) and 36.4% (276/759) if they had IV oxytocin (clinical scenario iii). This increased risk remained even after adjusting for prepregnancy and pregnancy factors (Table 4). Women who had IOL and epidural had twice the risk if they did not have IV oxytocin (2.06, [1.57‐2.69], *P* < 0.001) and 70% higher risk if they did have IV oxytocin (1.70 [CI 1.44‐2.01], *P* < 0.001). The above associations were very similar in the analysis that excluded women with multiple gestation, preterm gestation, or malpresentation.

**TABLE 4:** Clinical scenarios and the risk of unplanned CBs

Factor	Group	Unplanned CB (n = 722)	Vaginal birth (n = 1867)	ARR (95% CI)	*P*‐value
Case scenario	No IOL or IOL without epidural (with or without oxytocin)	377 (22.4%)	1304 (77.6%)	1 (Ref)	
	IOL, epidural, no oxytocin	66 (45.5%)	79 (54.5%)	2.06 (1.57‐2.69)	<0.001
	IOL, epidural, oxytocin	276 (36.4%)	483 (63.6%)	1.70 (1.44‐2.01)	<0.001
Age group	<25 years	29 (14%)	178 (86%)	0.59 (0.39‐0.89)	0.013
	25‐29 years	127 (24.2%)	397 (75.8%)	1 (Ref)	
	30‐34 years	292 (25.8%)	840 (74.2%)	0.99 (0.80‐1.23)	0.958
	35‐39 years	217 (34.8%)	406 (65.2%)	1.22 (0.96‐1.54)	0.098
	≥40 years	57 (55.3%)	46 (44.7%)	1.75 (1.25‐2.46)	0.001
Prepregnancy BMI	Ideal weight	403 (24.0%)	1275 (76.0%)	1 (Ref)	
	Overweight	178 (38.9%)	280 (61.1%)	1.57 (1.31‐1.88)	<0.001
	Obese/very obese	90 (35.7%)	162 (64.3%)	1.31 (1.04‐1.67)	0.025
	Missing	51 (25.4%)	150 (74.6%)	1.16 (0.85‐1.57)	0.360
Hypertension *M* = 41	No	664 (27.1%)	1783 (72.9%)	1 (Ref)	
	Yes	46 (45.5%)	55 (54.5%)	1.42 (1.04‐1.94)	0.026
Any diabetes *M* = 43	No	704 (27.8%)	1831 (72.2%)		
	Yes	7 (63.6%)	4 (36.4%)	1.61 (0.75‐3.42)	0.219
Asthma *M* = 28	No	568 (26.6%)	1567 (73.4%)	1 (Ref)	
	Yes	147 (34.5%)	279 (65.5%)	1.24 (1.03‐1.50)	0.022
Treatment for infertility *M* = 8	No	618 (26.5%)	1710 (73.5%)		
	Yes	102 (40.3%)	151 (59.7%)	0.99 (0.78‐1.25)	0.926
Type of care	Public	442 (25.7%)	1276 (74.3%)	1 (Ref)	
	Semiprivate	150 (28.4%)	379 (71.6%)	1.04 (0.86‐1.26)	0.672
	Private	130 (38.0%)	212 (62.0%)	1.28 (1.04‐1.59)	0.022
Number of fetus(es)	Single	695 (27.3%)	1851 (72.7%)	1 (Ref)	
	Multiple	27 (62.8%)	16 (37.2%)	1.57 (1.02‐2.41)	0.042
Gestational age	Term	650 (26.6%)	1796 (73.4%)	1 (Ref)	
	Preterm/very preterm	72 (50.4%)	71 (49.7%)	1.65 (1.25‐2.17)	<0.001
Cephalic presentation	Yes	664 (26.3%)	1864 (73.7%)	1 (Ref)	
	No	58 (95.1%)	3 (4.9%)	4.22 (3.16‐5.64)	<0.001

*Note*: Data were missing for clinical scenario (i) (n = 4), hypertension (n = 41), and asthma (n = 28). The model included prepregnancy (maternal age, prepregnancy BMI, and preexisting hypertension and asthma) and pregnancy (type of care, number of fetus(es), gestational age, and fetal presentation at birth) factors that were found to be significantly associated with an unplanned CB on bivariable analysis.

Abbreviations: ARR, adjusted risk ratio; BMI, body mass index; CI, confidence interval; IOL, induction of labor.

## 4 DISCUSSION

The risk of having a planned CB significantly increased for women who were aged ≥40 years, had treatment for infertility, were in semiprivate and private care, and had multiple gestation and breech or other malpresentation, after adjusting for the prepregnancy and pregnancy factors. Women aged ≥40 years, being overweight and obese/very obese prepregnancy, with preexisting hypertension or asthma, in private care, with multiple gestation and breech or other malpresentations, and who had IOL and epidural with or without the use of IV oxytocin in labor had a significantly increased risk of birthing by an unplanned CB, after controlling for prepregnancy and pregnancy factors.

In recent years, changing maternal characteristics and risk profiles, such as increasing maternal age and high BMI, treatment for infertility,^4^, ^10^, ^22^ are frequently reported as being associated with the increase in CBs. These resonate with our study with twofold increased risk of CB for women aged ≥40 years, one and half times increased risk for women with high BMI and doubled risk for women who had treatment for infertility. Although the change in maternal demographics partly contributes to the rising trend in CBs, this does not fully explain the overall decision‐making, and rising CB rates in nulliparous women.^4^ CB rates in many countries with low rates of CB have stayed at a 15%‐18% level for decades,^2^ despite an increase in average maternal age and obesity.^23^ The higher rates of CB for women with high BMIs can be attributed to the pathway followed for the management of their labor, involving a greater use of epidural and IV oxytocin in labor, and earlier decisions to perform CB in the second stage.^24^ This is well supported by clinicians' views from other studies that have stated that care of women with obesity is complex and challenging, with overmedicalization of intrapartum practices, and have suggested the need to challenge the current practice, promote normality, and optimize vaginal birth among obese women.^25^

Although limited by lack of data on whether women with breech presentation were offered ECV or were eligible to have ECV before the decision for a CB, a possible limitation of this study, women with fetal breech presentation and other malpresentations were at the highest risk for planned (16 times higher) and unplanned (four and half times higher) CBs consistent with findings from other studies.^4^, ^26^ Despite recommendations from the Health Service Executive (HSE) in Ireland to conduct vaginal breech births,^27^ the practice has remained unchanged in all the maternity units in Ireland. This can be attributed to changes in practice after the publication of findings from the term breech trial.^28^ Although the methodology and findings of the trial were critiqued,^29^ and long‐term outcomes of both the babies^30^ and mothers^31^ were shown to be similar in both arms of the trial, the results led to planned CB becoming the favored mode of birth for women with breech presentations. Recent studies have highlighted the need to re‐evaluate practices^14^ in order to reduce the number of planned CBs for fetal breech presentation.

There is wide variation in the strength of the relationship between IOL and unplanned CB with ongoing debate around the contribution of IOL to the rising rates of CB for low‐risk women. Two systematic reviews of randomized controlled trials in the past have concluded that IOL was associated with a reduced rate of CB among low‐risk women^32^, ^33^ in contrast to the findings of this study. The risk of CB in this study was twice as high for women whose labors were induced, and who used epidural analgesia, with or without IV oxytocin in labor. These findings are consistent with other large observational studies suggesting a doubled risk of unplanned CB with IOL among first‐time mothers^15^ illustrating the suspected consequences of the “cascade of intervention".^34^ Although we did not adjust for reasons or methods of IOL, reasons for and duration of use of IV oxytocin or epidural in labor, and possible limitations, previous studies have shown a significantly increased risk of CB regardless of the method of IOL^35^ and timing of epidural,^36^ and no difference in rates of CB irrespective of the time of onset of IV oxytocin for slow labor (early or late).^37^ Although our study findings do not report indication and time of administration of epidural, the findings suggest a significant association of epidural with CB for women whose labor was induced.

Although there is evidence around IOL being associated with reduced rates of CB (The ARRIVE trial)^38^, in contrast to the findings of the current study, there are recommendations to carefully consider translation of findings from the trial to clinical practice.^38^ However, the association between elements in the cascade of intervention in the current study highlights the need to revisit organizational guidelines on the criteria for selection of women for IOL, use of epidural analgesia and IV oxytocin for IOL, or augmentation of labor, and to introduce evidence‐based interventions^39^ designed to reduce CB. The resulting decrease in CBs for nulliparous women will also reduce the number of repeat CBs, with their concomitant ill effects on health‐related quality of life.^40^

Private care is one of the frequently reported factors associated with an increased risk of CB.^5^, ^6^, ^41^ Several studies have explored the underlying reasons for this association. Decision‐making in private practice, in some countries, is often described as being related to pay and reimbursement system, the financial incentives associated with CB, and benefits to the consultants; others have attributed it to continuity of care and women being inclined to go with the flow of their consultant obstetricians' recommendation, or clinicians' “convenience,”^41^ or women being able to afford health care insurance to choose private and continuity of care by a senior obstetrician.^6^ Findings from this study resonate with what has been reported previously^41^ suggesting a significantly increased risk of both planned and unplanned CBs for women in private care compared with women in public care.

The study findings are possibly limited due to the potential for recruitment bias since the questionnaires were available in English only, which precluded the recruitment of women who did not read or understand English. This proportion is not known due to a lack of data on the use of interpreter services at the time of recruitment. However, the strength of this study lies in its uniqueness of presenting results from 3047 first‐time mothers recruited in early pregnancy from the three (two large‐ and one moderate‐sized) maternity hospitals in the Republic of Ireland, which enhanced the generalizability of the findings. Survey data collected prospectively from women in early pregnancy and the high response rate (84% of the total women eligible for follow‐up at 3 months postpartum), combined with clinician‐reported data collected from hospital records of consenting women, add strength to the findings. Although findings are not adjusted for reasons, length or methods of IOL, and IV oxytocin or epidural, which is a possible limitation, presentation of the risk of having an unplanned CB in the common clinical scenarios and intrapartum interventions added to the discussion of factors associated with CB.

### 4.1 Conclusions

There are multiple factors associated with the risk of CB. Understanding these factors has the potential to help identify possible explanations for the rising trend in CB among first‐time mothers with the goal of reducing any inappropriate CBs and repeat CBs in future pregnancies. Given the evidence around a significant association between IOL (with other prelabor and labor interventions such as epidural and IV oxytocin) and unplanned CBs, there is an opportunity to address practices around these interventions in order to avoid any unnecessary CBs. This can be achieved through revision of policies, clinical protocols, and guidelines, such as criteria for IOL and offering/performing ECV, conducting regular audits of clinical practice and birth outcomes to ensure adherence to the guidelines for women in all models of care, public and private. Based on the identified risk factors, there is potential for clinicians to develop strategies to reduce CBs safely in nulliparous women, ultimately leading to a reduction in the number of repeat CBs in multiparous women.

## CONFLICT OF INTERESTS

The authors have no conflicts of interest.

## Supporting information


Table S1
Table S2
Table S3
Table S4

## Data Availability

The data that support the findings of this study are available on request from the corresponding author. The data are not publicly available due to privacy or ethical restrictions.

## References

[1] Betrán AP , Ye J , Moller A‐B , Zhang J , Gülmezoglu AM , Torloni MR . The increasing trend in caesarean section rates: global, regional and National Estimates: 1990‐2014. PloS One. 2016;11(2):e0148343. doi:10.1371/journal.pone.0148343 26849801 PMC4743929

[2] Euro‐Peristat Project . European Perinatal Health Report. Core indicators of the health and care of pregnant women and babies in Europe in 2015. 2018. http://www.europeristat.com. Accessed March 10, 2021.

[3] McMahon L , McGrane K , McKenna P , Turner M . National Women and Infants Health Programme (NWIHP) Clinical Programme for Obstetrics and Gynaecology. Irish Maternity Indicator System (IMIS) National Report 2019. National Women and Infants. Health Programme for Obstetrics and Gynaecology, Ireland. 2020. https://www.hse.ie/eng/about/who/acute‐hospitals‐division/woman‐infants/national‐reports‐on‐womens‐health/imis‐national‐report‐2019.pdf. Accessed March 10, 2021.

[4] Brick A , Layte R , Nolan A , Turner M . Differences in nulliparous caesarean section rates across models of care: a decomposition analysis. BMC Health Serv Res. 2016;16(239):1‐12. doi:10.1186/s12913-016-1494-3 27392410 PMC4938942

[5] Moran PS , Daly D , Wuytack F , et al. Predictors of choice of public and private maternity care among nulliparous women in Ireland, and implications for maternity care and birth experience. Health Policy. 2020;124(5):556‐562. doi:10.1016/j.healthpol.2020.02.008 32284156

[6] Turner MJ , Reynolds C , McMahon LE , O’Malley EG , O’Connell MP , Sheehan SR . Caesarean section rates in women in the Republic of Ireland who chose to attend their obstetrician privately: a retrospective observational study. BMC Pregnancy Childbirth. 2020;20(1):548. doi:10.1186/s12884-020-03199-x 32957947 PMC7504647

[7] Souza JP , Gülmezoglu AM , Lumbiganon P , et al. Caesarean section without medical indications is associated with an increased risk of adverse short‐term maternal outcomes: the 2004‐2008 WHO global survey on maternal and perinatal health. BMC Med. 2010;8(1):71. 21067593 10.1186/1741-7015-8-71 PMC2993644

[8] Keag OE , Norman JE , Stock SJ . Long‐term risks and benefits associated with cesarean delivery for mother, baby, and subsequent pregnancies: systematic review and meta‐analysis. PLoS Med. 2018;15(1):e1002494. doi:10.1371/journal.pmed.1002494 29360829 PMC5779640

[9] Muto H , Ishii K , Nakano T , Hayashi S , Okamoto Y , Mitsuda N . Rate of intrapartum cesarean section and related factors in older nulliparous women at term. J Obstet Gynaecol Res. 2018;44(2):217‐222. doi:10.1111/jog.13522 29094431

[10] Renes L , Barka N , Gyurkovits Z , Paulik E , Nemeth G , Orvos H . Predictors of caesarean section ‐ a cross‐sectional study in Hungary. J Maternal‐Fetal Neonat Med. 2017;31:1‐5. doi:10.1080/14767058.2017.1285888 28110607

[11] Rydahl E , Declercq E , Juhl M , Maimburg RD . Cesarean section on a rise‐does advanced maternal age explain the increase? A population register‐based study. PLOS One. 2019;14(1):1‐16. doi:10.1371/journal.pone.0210655 PMC6345458 30677047

[12] Chauhan SP , Beydoun H , Hammad IA , et al. Indications for caesarean sections at ≥34 weeks among nulliparous women and differential composite maternal and neonatal morbidity. Br J Obstet Gynaecol. 2014;121:1395‐1402. doi:10.1111/1471-0528.12669 24506582

[13] Carbillon L , Benbara A , Tigaizin A , et al. Revisiting the management of term breech presentation: a proposal for overcoming some of the controversies. BMC Pregnancy Childbirth. 2020;20(1):263. doi:10.1186/s12884-020-2831-4 32359354 PMC7196223

[14] Kacerauskiene J , Minkauskiene M , Mahmood T , et al. Lithuania’s experience in reducing caesarean sections among nulliparas: the impact of the quality improvement course. BMC Pregnancy Childbirth. 2020;20(1):152. doi:10.1186/s12884-020-2806-5 32164550 PMC7069017

[15] Davey MA , King J . Caesarean section following induction of labour in uncomplicated first births‐ a population‐based cross‐sectional analysis of 42,950 births. BMC Pregnancy Childbirth. 2016;16(1):92. doi:10.1186/s12884-016-0869-0 27121614 PMC4848820

[16] Uno K , Mayama M , Yoshihara M , et al. Reasons for previous cesarean deliveries impact a woman’s independent decision of delivery mode and the success of trial of labor after cesarean. BMC Pregnancy Childbirth. 2020;20(1):170. doi:10.1186/s12884-020-2833-2 32204702 PMC7092517

[17] Daly D , Clarke M , Begley C . Urinary incontinence in nulliparous women before and during pregnancy: prevalence, incidence, type, and risk factors. Int Urogynecol J. 2018;29(3):353‐362. doi:10.1007/s00192-018-3554-1 29362836

[18] Healthcare Pricing Office . Perinatal Statistics Report 2016. Health Service Executive (HSE); 2018. http://www.hpo.ie/latest_hipe_nprs_reports/NPRS_2016/Perinatal_Statistics_Report_2016.pdf. Accessed March 10, 2021.

[19] Malone F. Annual clinical report. The rotunda hospital, Dublin, Ireland; 2017. https://rotunda.ie/rotunda‐annual‐report‐2017 Accessed July 12, 2018.

[20] Ryan E. (2017) Annual Clinical Report. The Galway University Hospital, Ireland. https://saolta.ie/sites/default/files/publications/Saolta_Annual_Report_2017.pdf, Accessed December 12, 2018.

[21] Sheehan S. (2017) Annual Clinical Report. The Coombe Women and Infants University Hospital. http://www.coombe.ie/index.php?nodeId=110 Accessed May 12, 2018.

[22] Brick A , Layte R , McKeating A , Sheehan SR , Turner MJ . Does maternal obesity explain trends in caesarean section rates? Evidence from a large Irish maternity hospital. Ir J Med Sci. 2020;189(2):571‐579. doi:10.1007/s11845-019-02095-4 31591684

[23] Euro Peristat Fertility Indicators . The Statistical Office of the European Union. The Statistical Office of the European Union; 2019.

[24] Abenhaim H , Benjamin A . Higher caesarean section rates in women with higher body mass index: are we managing labour differently? J Obstet Gynaecol Canada. 2011;33(5):443‐448. doi:10.1016/S1701-2163(16)34876-9 21639963

[25] Kerrigan A , Kingdon C , Cheyne H . Obesity and normal birth: a qualitative study of clinician’s management of obese pregnant women during labour. BMC Pregnancy Childbirth. 2015;15:256. doi:10.1186/s12884-015-0673-2 26459259 PMC4603577

[26] Colomar M , Cafferata ML , Aleman A , et al. Mode of childbirth in low‐risk pregnancies: Nicaraguan physicians' viewpoints. Matern Child Health J. 2014;18(10):2382‐2392. doi:10.1007/s10995-014-1478-z 24740720

[27] Health Service Executive . In: Executive HS , ed. eds. IrelandNATIONAL CLINICAL GUIDELINE ‐ the Management of Breech Presentation. Health Service Executive; 2017. https://www.hse.ie/eng/about/who/acute‐hospitals‐division/woman‐infants/clinical‐guidelines/the‐management‐of‐breech‐presentation.pdf. Accessed March 10, 2021.

[28] Hannah ME , Hannah WJ , Hewson SA , Hodnett ED , Saigal S , Willan AR . Planned caesarean section versus planned vaginal birth for breech presentation at term: a randomised multicentre trial. Term breech trial collaborative group. Lancet. 2000;356(9239):1375‐1383. doi:10.1016/s0140-6736(00)02840-3 11052579

[29] Lawson GW . The term breech trial ten years on: primum non Nocere? Birth. 2012;39(1):3‐9. doi:10.1111/j.1523-536X.2011.00507.x 22369600

[30] Whyte H , Hannah ME , Saigal S , et al. Term breech trial collaborative group: outcomes of children at 2 years after planned cesarean birth versus planned vaginal birth for breech presentation at term: the international randomized term breech trial. Am J Obstet Gynecol. 2004;191:864‐871. doi:10.1016/j.ajog.2004.06.056 15467555

[31] Hannah ME , Whyte H , Hannah WJ , et al. Term breech trial collaborative group: maternal outcomes at 2 years after planned cesarean section versus planned vaginal birth for breech presentation at term: the international randomized term breech trial. Am J Obstet Gynecol. 2004;191:917‐927. doi:10.1016/j.ajog.2004.08.004 15467565

[32] Mishanina E , Rogozinska E , Thatthi T , Uddin‐Khan R , Khan KS , Meads C . Use of labour induction and risk of cesarean delivery: a systematic review and meta‐analysis. CMAJ. 2014;186(9):665‐673. doi:10.1503/cmaj.130925 24778358 PMC4049989

[33] Saccone G , Berghella V . Induction of labor at full term in uncomplicated singleton gestations: a systematic review and meta‐analysis of randomized controlled trials. Am J Obstet Gynecol. 2015;213(5):629‐636. doi:10.1016/j.ajog.2015.04.004 25882916

[34] Tracy SK , Sullivan E , Wang YA , Black D , Tracy M . Birth outcomes associated with interventions in labour amongst low risk women: a population‐based study. Women Birth. 2007;20(2):41‐48. doi:10.1016/j.wombi.2007.03.005 17467355

[35] Thorsell M , Lyrenäs S , Andolf E , Kaijser M . Induction of labor and the risk for emergency cesarean section in nulliparous and multiparous women. Acta Obstet Gynecol Scand. 2011;90(10):1094‐1099. doi:10.1111/j.1600-0412.2011.01213.x 21679162

[36] Klein MC . Does epidural analgesia increase rate of cesarean section? Can Fam Physician. 2006;52(4):419‐428. 16639961 PMC1481670

[37] Bugg GJ , Siddiqui F , Thornton JG . Oxytocin versus no treatment or delayed treatment for slow progress in the first stage of spontaneous labour. Cochrane Database Syst Rev. 2013;6:1465‐1858. doi:10.1002/14651858.CD007123.pub3 PMC11836144 23794255

[38] Carmichael SL , Snowden JM . The ARRIVE trial – interpretation from an epidemiologic perspective. J Midwifery Womens Health. 2019;64(5):657‐663. doi:10.1111/jmwh.12996 31264773 PMC6821557

[39] Smith V , Gallagher L , Carroll M , Hannon K , Begley C . Antenatal and intrapartum interventions for reducing caesarean section, promoting vaginal birth, and reducing fear of childbirth: an overview of systematic reviews. Plos One. 2019;14(10):e0224313. doi:10.1371/journal.pone.0224313 31648289 PMC6812784

[40] Fobelets M , Beeckman K , Buyl R , et al. Mode of birth and postnatal health‐related quality of life after one previous cesarean in three European countries. Birth. 2018;45(2):137‐147. doi:10.1111/birt.12324 29205463

[41] Panda S , Begley C , Daly D . Clinicians' views of factors influencing decision‐making for caesarean section: a systematic review and metasynthesis of qualitative, quantitative and mixed methods studies. PloS One. 2018;13(7):e0200941. doi:10.1371/journal.pone.0200941 30052666 PMC6063415

