# Supplementary material for: Factors associated with cesarean birth in nulliparous women: A multicenter prospective cohort study
Source: Birth. 2022 Jun 13;49(4):812–22. doi: 10.1111/birt.12654 (PMC9796356; doi:10.1111/birt.12654)
Supplement: Supplementary file 1 — Table S1 Table S2 Table S3 Table S4 [file BIRT-49-812-s001.docx]

**Supplementary Table S1 - Socio-demographic characteristics of women in the study**

| **Socio-demographic characteristics** | **Frequency** | | **%** |
| --- | --- | --- | --- |
| **Maternal age (n=3047)** |  |  | |
| Up to 24 years | 239 | 7.8 | |
| 25-29 years | 620 | 20.4 | |
| 30-34 years | 1316 | 43.2 | |
| 35-39 years | 734 | 24.1 | |
| 40 years & over | 138 | 4.5 | |
| **Pre-pregnancy BMI (n=3047)** |  |  | |
| Ideal weight (≤24.9kg/m^2^) | 1974 | 64.8 | |
| Overweight (25-29.99kg/m^2^) | 539 | 17.7 | |
| Obese/very obese (≥30 kg/m^2^) | 287 | 9.4 | |
| Missing | 247 | 8.1 | |
| **Ethnicity (n=3045)** |  |  | |
| Irish | 2168 | 71.2 | |
| Irish traveller | 2 | 0.1 | |
| African | 49 | 1.6 | |
| Chinese | 13 | 0.4 | |
| Any other white background | 716 | 23.5 | |
| Any other black background | 4 | 0.1 | |
| Any other Asian background | 63 | 2.1 | |
| Other including mixed background | 30 | 1 | |
| Missing | 2 |  | |
| **Educational qualification (n=2987)** |  |  | |
| Degree/postgraduate degree | 2032 | 68 | |
| Diploma, Cert, or equivalent | 565 | 18.9 | |
| Up to secondary level | 390 | 13.1 | |
| Missing | 60 |  | |
| **Employment status in early pregnancy (n=3000)** |  |  | |
| Employed | 2636 | 87.9 | |
| Unemployed | 206 | 6.9 | |
| Other | 158* | 5.3 | |
| Not stated/not applicable | - | - | |
| Missing | 47 |  | |
| **Relationship status (n=2996)** |  |  | |
| Married | 1828 | 61 | |
| Single | 92 | 3.1 | |
| In relationship with or without partner | 1046 | 34.9 | |
| Other (Divorced, widowed, separated) | 30 | 1 | |
| Missing | 51 |  | |

**Voluntary job, student.*

**Supplementary Table S2 - Pregnancy and birth details of participants**

| **Pregnancy and birth details** | **Frequency**  **(n=2755)** | | **%** |
| --- | --- | --- | --- |
| **Treatment for infertility** |  |  | |
| No treatment | 2452 | 89.3 | |
| Treatment for infertility (Fertility drugs, IVF/ICSI, other) | 294 | 10.7 | |
| Missing | 9 |  | |
| **Type of care** |  |  | |
| Public | 1795 | 65.2 | |
| Semi-private | 574 | 20.8 | |
| Private | 386 | 14 | |
| **Number of fetus(es)** |  |  | |
| Singleton gestation | 2700 | 98 | |
| Multiple gestation | 55 | 2 | |
| **Gestational age at birth** |  |  | |
| Term | 2594 | 94.2 | |
| Preterm and very preterm | 161 | 5.8 | |
| **Presentation of fetus at birth** |  |  | |
| Cephalic | 2619 | 95.1 | |
| Breech and other malpresentations | 136 | 4.9 | |
| **Induction of labour (IOL)** |  |  | |
| No IOL | 1655 | 60.3 | |
| IOL | 1089 | 39.7 | |
| Missing | 11 |  | |
| **Intravenous oxytocin** |  |  | |
| Labour without oxytocin | 1325 | 48.3 | |
| Labour with oxytocin | 1417 | 51.7 | |
| Missing | 13 |  | |
| **Epidural for pain management in labour** |  |  | |
| No epidural | 566 | 26.7 | |
| Epidural | 1554 | 73.3 | |
| Missing | 635 |  | |
| **Mode of birth** |  |  | |
| SVB | 926 | 33.6 | |
| AVB | 941 | 34.2 | |
| Planned CB | 166 | 6 | |
| Unplanned CB | 722 | 26.2 | |

**Supplementary Table S3 – Reasons for panned and unplanned CB**

| **Reasons for planned CB** | **Frequency** | **%** |
| --- | --- | --- |
| Breech presentation | 74 | 44.6 |
| Maternal request | 13 | 7.8 |
| Preeclampsia/Pregnancy induced hypertension | 6 | 3.6 |
| Placenta previa | 6 | 3.6 |
| Unstable lie | 2 | 1.2 |
| Not indicated | 5 | 3 |
| Other fetal reasons* | 37 | 22.3 |
| Other maternal reasons** | 23 | 13.9 |
| Total | 166 | 100 |
| Reasons for unplanned CB | Frequency | % |
| Fetal distress | 337 | 46.7 |
| Lack of progress in first stage of labour | 78 | 10.8 |
| Lack of progress in second stage of labour | 77 | 10.7 |
| Failed IOL | 65 | 9 |
| Fetal breech presentation in labour | 44 | 6.1 |
| Other maternal reasons*** | 35 | 4.9 |
| Hypertension/pregnancy induced hypertension/ Preeclampsia/HELPP syndrome | 26 | 3.6 |
| Antepartum haemorrhage/abruption/placenta previa | 22 | 3.1 |
| Other fetal reasons**** | 17 | 2.4 |
| Unstable lie | 3 | 0.4 |
| Not indicated | 18 | 2.5 |
| Total | 722 | 100 |

*Other fetal reasons included: big baby (n=13), high vertex (n=13), reduced fetal growth (n=7), fetal anomaly (n=2), absent end diastolic flow (n=1), triplets (n=1). **Other maternal reasons included infectious disease (genital herpes, HSV, HIV) (n=3), perforated uterus (n=3), fractured pelvis/hip replacement (n=3), cardiac condition (n=2), retinal detachment (n=2), previous myomectomy (n=2), maternal age/treatment for infertility (n=1), bleeding disorder (n=1), anal fistula (n=1), ovarian cyst (n=1), Ashermann’s syndrome (n=1), past poor obstetric history (n=1), diabetes (n=1), increased liquor volume (n=1).

***Other maternal reasons included: pelvic pain/spinal problems (n=8), previous myomectomy (n=5), bleeding disorder (n=4), past poor obstetric history (n=4), other medical conditions (n=2), anal fistula/rectal prolapse (n=2), maternal request for social reasons (n=2), hyperstimulation with oxytocin (n=1), corneal ectopic (n=1), infectious disease (Genital herpes) (n=1), maternal age/treatment for infertility (n=1), fibroid (n=1), baby in occipito posterior position and postdate pregnancy (n=1), maternal pyrexia (n=1), increased liquor volume (n=1)

****Other fetal reasons included reduced fetal growth/reduced liquor volume (n=5), multiple gestation (n=4), high vertex (n=3), big baby (n=2), fetal malposition (n=2), cord prolapsed (n=1).

*IOL – Induction of Labour*

*HELLP – High blood pressure Elevated Liver Enzymes and Low Platelets*

**Supplementary Table S4 – Reasons for IOL**

| **Reasons for IOL** | **Frequency (%)** | **CB** |
| --- | --- | --- |
| Post-term gestation (≥37 weeks) | 341 (31.3%) | 133/341 (39%) |
| Any diabetes | 58 (5.3%) | 24/58 (41.4%) |
| Prolonged rupture of membranes | 242 (22.2%) | 79/242 (32.6%) |
| Pregnancy induced hypertension/Preeclampsia | 109 (10%) | 36/109 (33%) |
| Big baby | 26 (2.4%) | 13/26 (50%) |
| Fetal distress | 2 (0.2%) | 1/2 (50%) |
| Increased liquor volume | 2 (0.2%) | 1/2 (50%) |
| Maternal age | 7 (0.6%) | 3/7 (42.9%) |
| Reduced fetal growth | 59 (5.4%) | 15/59 (25.4%) |
| Reduced liquor volume | 49 (4.5%) | 19/49 (38.8%) |
| Obstetric cholestasis | 30 (2.8%) | 7/30 (23.3%) |
| Reduced fetal movements | 25 (2.3%) | 7/25 (28%) |
| Antepartum haemorrhage | 24 (2.2%) | 6/24 (25%) |
| Other maternal reasons* | 16 (1.5%) | 1/16 (6.3%) |
| Multiple gestation | 10 (0.9%) | 3/10 (30%) |
| Social reasons | 7 (0.6%) | 2/7 (28.6%) |
| Other fetal reasons** | 5 (0.5%) | 1/5 (20%) |
| Pregnancy following treatment for infertility | 3 (0.3%) | 0 |
| Not indicated | 74 (6.8%) | 39/74 (52.7%) |
| Total | 1089 (100%)*** | 390/1089 (35.8%) |

*Other maternal reasons included pelvic pain (n=4), nephrotomy/hydronephrosis (n=2), maternal distress (n=1), anxiety (n=1), cardiac murmur (n=1), mature placenta (n=1), past poor obstetric history (n=1), proteinuria (n=1), gall stones (n=1), epilepsy (n=1), removal of cervical suture (n=1), history of cancer (n=1).

**Other fetal reasons included fetal anomaly (n=2), cystic hygroma (n=1), encephalocele (n=1), Triosomy 21 (n=1).

*** For two women who had an IOL it is not known if they had received an epidural or oxytocin.
